# ‘Genetic Coding’ Reconsidered: An Analysis of Actual Usage

**DOI:** 10.1093/bjps/axv007

**Published:** 2015-03-15

**Authors:** Ulrich E. Stegmann

**Affiliations:** School of Divinity History and Philosophy University of Aberdeen Aberdeen, UK

## Abstract

This article reconsiders the theoretical role of the genetic code. By drawing on published and unpublished sources from the 1950s, I analyse how the code metaphor was actually employed by the scientists who first promoted its use. The analysis shows that the term ‘code’ picked out mechanism sketches, consisting of more or less detailed descriptions of ordinary molecular components, processes, and structural properties of the mechanism of protein synthesis. The sketches provided how-possibly explanations for the ordering of amino acids by nucleic acids (the ‘coding problem’). I argue that employing the code metaphor was justified in virtue of its descriptive-denotational and explanatory roles, and because it highlighted a similarity with conventional codes that was particularly salient at the time.

**1** *Introduction*
**2** *Coding Schemes in the 1950s*
    **2.1** *The research problem: Determining amino acid sequences*
    **2.2** *The solution: Mapping schemes or ‘codes’*
**3** *The Code Metaphor Played Descriptive and Explanatory Roles*
**4** *The Abstractness of Codes and the Expendability of the Code Metaphor*
**5** *The Role of Arbitrariness*
**6** *Conclusions*

## 1 Introduction

The ‘genetic code’ is perhaps one of the most familiar metaphors in biology. At one level the metaphor is nowadays simply a label for a set of causal relations important for protein synthesis, namely, the mapping between RNA base triplets and amino acids. But the widespread use of semantic language in molecular biology, as exemplified by ‘code’, is controversial among historzians and philosophers of biology (for example, Sarkar [1996]; Godfrey-Smith [2000]; Kay [2000]; Griffiths [2001]). At stake is the question of whether using apparently semantic concepts is legitimate and whether they should be taken literally, indicating the presence of some semantic or proto-semantic properties in a domain of science that is otherwise solidly grounded in physics and chemistry. With respect to the genetic code, for instance, some have argued that its use is inappropriate and misleading (Kay [2000]), while others have defended it as well-motivated (for example, Godfrey-Smith [2000]; Maynard Smith [2000]).

In these discussions, cognate concepts like genetic code and genetic information are often treated together without paying much attention to possible differences. More importantly, despite a wealth of historical sources and a rich historiography of early research into protein synthesis (for example, de Chadarevian [1996]; Judson [1996]; Rheinberger [1997]; Kay [2000]), we still know very little about how the scientists themselves used the code metaphor in practice, especially at the time when it was introduced and began to be employed in a sustained way. Yet a detailed analysis of actual usage at that time could be revealing. It could show (i) to what ‘code’ referred, if anything, (ii) what the properties of its referent were, (iii) whether these properties included arbitrariness and in some sense semantic features, and (iv) whether it played valuable theoretical roles. Answers to these questions will allow historical conclusions about a key episode in the history of molecular genetics. But they will also have philosophical import. If it can be shown, for example, that the code metaphor played specific theoretical roles in the emerging science of molecular biology, then its use could be justified by appeal to these roles. It may also turn out that the referent of ‘code’ bore certain similarities with human symbol systems, which could again motivate its application to molecular biology.

The aim of this article is to provide such an analysis and draw the relevant philosophical conclusions. The analysis is based on a close reading of bothpublished material and unpublished sources by the proponents of non-experimental research into protein synthesis in the 1950s (for example, Francis Crick, George Gamow, James Watson, and their co-workers). In recent years, unpublished material has become readily available via online archives.^1^ Some restrictions were necessary in order to keep the project manageable. First, the analysis of actual usage is limited to the period between 1953 and 1958, which saw the first steps towards elucidating protein synthesis and the gradual consolidation of the code metaphor. Second, the analysis is restricted to the code metaphor, putting aside cognate notions like genetic information. Third, I focus on the possible descriptive and explanatory roles of the code metaphor, bracketing potential predictive and heuristic uses (to be explored elsewhere).

The article is organized as follows: Section 2 presents the results of the analysis of actual usage. Section 3 argues that the code concept picked out mechanism sketches and thus played useful descriptive and explanatory roles.Section 4 refines these claims by addressing the alleged abstractness of coding schemes and the expendability of the code concept. In Section 5 I address the (in)significance of arbitrariness for understanding the status of coding.

## 2 Coding Schemes in the 1950s

### 2.1 The research problem: Determining amino acid sequences

Biochemists recognized protein synthesis as a research problem since at least the 1940s (Judson [1996]; Rheinberger [1997]). They also acknowledged that one of its crucial aspects was arranging amino acids into linear sequences. By 1953, two groups of hypotheses had emerged to explain this process (Campbell and Work [1953]). According to the first group, protein-specific amino acid sequences resulted from a series of highly specific enzymatic reactions: enzymes would catalyse the linkage between two adjacent amino acids, with different enzymes responsible for conjoining different types of amino acids (for example, ‘transpeptidation theory’). According to the second group of hypotheses, new amino acid chains were the result of free amino acids attaching temporarily to pre-existing protein or nucleic acid templates (for example, Haurowitz [1950]; Dounce [1952]).

Efforts to understand protein synthesis were re-invigorated by Watson and Crick’s ([1953a]) double helix model of DNA. The model allowed innumerable permutations of bases along its sugar-phosphate backbone and, hence, the possibility of it being responsible for the order of amino acids in proteins (Olby [1994], pp. 427, 434; Judson [1996], p. 280). George Gamow, a cosmologist, was quick to explore this possibility. He developed the first model of protein synthesis based on Watson and Crick’s structure. When in October 1953 he submitted his manuscript to *Nature* (published as Gamow [1954a]), he wrote to Linus Pauling:

Ever since I read the article of Watson and Crick last June, I was trying to figure out how a long number written in a fourdigital system (i.e. nucleic acid molecule) can determine unickly [sic] a correspondingly long word based on [a] 20-letter-alphabet (i.e. an enzime [sic] molecule). (Gamow [1953])

Implicit in Gamow’s quote is a hypothesis about the ‘determination’ of amino sequence that had been anticipated by earlier template models: the amino acidorder in proteins is determined solely by the linear order of discrete elements along the template, rather than by the template’s three-dimensional shape.^2^ Watson made the last point explicit in handwritten notes: ‘[w]e believe [the] secondary structure to be irrelevant to the main argument’ (Watson [1954–55]). The ‘secondary structure’ referred to the conformation (rather than base sequence) of the nucleic acid template and ‘the main argument’ to ‘the determination of sequence’ of amino acids by nucleic acids. A few years later, Crick ([1958a]) articulated this idea as part of his ‘sequence hypothesis’.

The assumption that the amino acid sequence only depends on the base sequence simplified the task of understanding protein synthesis because it allowed the bracketing of a possible role for the three-dimensional conformation of nucleic acids. The task became to understand how a nucleic acid sequence could order incoming amino acids into a linear chain. Since nucleic acids were thought to be composed of four types of bases and polypeptides of twenty kinds of amino acids,^3^ there were too few bases for each type of base to select one type of amino acid. There would have to be some other way by which nucleic acids can order amino acids. Scientists like Crick, Gamow, Watson, Brenner, and their co-workers agreed both on the nature of this task and the key assumption underlying it. Most of Gamow’s, Crick’s, and Brenner’s publications on protein synthesis between 1954 and 1958 contain opening comments that state or imply both points (for example, Gamow [1954a]; Gamow *et al.* [1956]; Brenner [1957]; Crick [1958a]). Crick articulated them explicitly:

[…] the order of the amino acids is determined by the order of the nucleotides of the nucleic acid. There are some twenty naturally occurring amino acids commonly found in proteins, but (usually) only four different nucleotides. The problem of how a sequence of four things (nucleotides) can determine a sequence of twenty things (amino acids) is known as the ‘coding’ problem. (Crick *et al.* [1957], p. 416)

### 2.2 The solution: Mapping schemes or ‘codes’

The coding problem was addressed by schemes that attempted to explain how a sequence of four types of bases can determine a sequence of twenty aminoacids. All schemes shared some core features: they all hypothesized thatnucleic acid chains consisted of strings of smaller units with the causal disposition to select one type of amino acid to be added to the growing peptide.

The period between 1953 and 1958 saw a bewildering variety of such hypothetical units and their relations with amino acids (Tables 1 and 2). A big divide existed between ‘overlapping’ and ‘non-overlapping’ schemes (Crick [1955]; Ledley [1955]; Brenner [1957]; Crick *et al.* [1957]; Gamow and Yčas [1958]; Golomb *et al.* [1958]). In non-overlapping schemes, all the individual components of a template unit (for example, the bases C, G, and A, in the unit CGA) were taken to belong to that unit only, and the unit selected only one amino acid while exerting no influence on the addition of the preceding or following amino acid. In overlapping schemes, by contrast, the components of a given template unit were part of the preceding or following unit (for example, the token base A in the unit CGA would be part of the next unit GAT).

**Table 1.** Overlapping mapping schemes that were labelled ‘codes’ (introduced between 1953 and 1958)

Name	‘Diamond’	‘Triangular’	‘Major-Minor’	‘Sequential’	‘Combination’	

Introduced by	Gamow ([1954a])	Gamow [1954d]	Gamow, Watson, Rich, and Orgel (Gamow [1954e]); Orgel (Gamow *et al.* [1956], p. 51)	Teller (Yčas [1954])	Crick ([1955], p. 10)	
Labelled ‘code’ by	Yčas ([1954]); Gamow (Gamow [1955], p. 2; Gamow *et al.* [1956], p. 41); Crick ([1955], p. 2); Brenner ([1957], p. 687)	Gamow (Gamow [1954d]; Gamow *et al.* [1956], p. 48); Yčas ([1954])	Gamow (Gamow [1954e]; Gamow *et al.* [1956], p. 51); Brenner ([1957], p. 687)	Yčas ([1954]); Crick ([1955], p. 16); Gamow *et al.* ([1956], p. 53)	Crick ([1955], p. 10)	
Degeneracy	Present; Varying (Crick [1955], p. 10)	Present; NS	Present; NS	NS	Present; varying	
Attachment	Directly	Directly	Directly	Directly	Indirect [?] (Crick [1955], pp. 9–10)	
Directionality	Absent (Crick [1955], p. 11)	NS	NS	NS	Absent (Crick [1955], p. 12)	
Template	DNA	DNA or RNA	RNA	2 NA and previous AA	DNA or RNA (Crick [1955], p. 16)	
Single/Double	Double	Single	Single	NS	NS	
One/Both	Both	One	One	NS	NS	
Cardinality	4 bases (in diamonds)	3 bases (in triangles)	3 bases	2 bases + 1 AA	3 bases	

Name	‘Easy-Neighbour’	‘Directional’	(Crick ([1955], p. 15)	(Crick [1955], pp. 15–16)^13^	(Crick ([1955], pp. 13–14)	‘Dyad code’^14^

Introduced by	Crick ([1955], p. 11)	Crick ([1955], p. 12)	Crick ([1955], p. 15)	Crick ([1955], p. 16)	Crick ([1955], p. 13–14)	Dounce ([1958])
Labelled ‘code’ by	Crick ([1955], p. 11)	Crick ([1955])	Crick ([1955])	Crick ([1955])	Crick ([1955], p. 13–14)	Crick ([1958b])
Degeneracy	Present; uniform	Present; both possible	Present; both possible [?]	Present; both possible [?]	Present; varying	Present
Attachment	Indirect [?] (Crick [1955], pp. 9–10)	Indirect [?] (Crick [1955], pp. 9–10)	Via adaptor	Via adaptor	NS	NS
Directionality	Absent (Crick [1955], p. 12)	Present	NS	NS	Absent (Crick [1955], p. 14)	NS
Template	DNA or RNA (Crick [1955], p. 16)	DNA or RNA (Crick [1955], p. 16)	DNA or RNA (Crick [1955], p. 16)	DNA or RNA (Crick [1955], p. 16)	DNA or RNA (Crick [1955], p. 16)	RNA
Single/Double	NS	NS	Double	Double	Double	Single
One/Both	NS	NS	One	Both	Both	One
Cardinality	3 bases	3 bases	3 bases	3 bases	4 bases (diamond)	2 bases

‘NS’ here means ‘not specified’. ^13^ Described as ‘a special type of our wide class considered earlier’ (Crick [1955], p. 16), which may refer to the scheme on pp. 13–14. ^14^ As inferred from the correspondence between Dounce and Crick; Dounce’s manuscript was unavailable. Each of the two bases in a dyad can occur in eight states, because Dounce assumed that each of the four RNA bases could occur in an ‘up’ or ‘down’ position.

**Table 2.** Non-overlapping mapping schemes that were labelled ‘codes’ (introduced until 1958)

Name	‘Combination’^15^	‘Quadruplet’^16^	‘Comma-less’	(Simon)	(Szilard)^17^	Actual code

Introduced by	Gamow and Yčas ([1955])	Crick ([1956]); Crick and co-workers (Crick [1957b])	Crick *et al.* ([1957])	Simon (Yčas [1958], p. 92)	Szilard (Szilard [1957]; Kay [2000], p. 168)	Not proposed prior to empirical findings in the 1960s
Labelled ‘code’ by	Drake and Alderson ([1956]); Crick *et al.* ([1957], p. 420); Yčas ([1958], p. 92)	Crick ([1956], [1957b])	Crick *et al.* ([1957]); Delbrück (Golomb *et al.* [1958])	Yčas ([1958], p. 92)	Crick ([1957a])	
Comma	NS	Absent	Absent	NS	NS	Absent
Degeneracy	Present; varying	NS	Absent	Present; NS	NS	Present; varying
Attachment	Directly (Drake and Alderson [1956], p. 4)	NS	Directly or indirectly	NS	Indirectly	Via adaptor (tRNA)
Directionality	NS	Present	NS	NS	NS	Absent
Template	RNA	RNA or DNA	RNA (or DNA)	RNA	RNA	RNA
Single/Double	Single (Yčas [1958], p. 92)	Both possible	Single (if RNA)	Double	Single	Single
One/Both	One (Yčas [1958], p. 92)	Both possible	One (if RNA)	Both	One	One
Cardinality	3 bases	4 bases	3 bases	6 bases (i.e. 3 pairs)	Mix of 3- and 4-bases units	3 bases
Synthesis	NS	NS	Simultaneously	NS	Successively	Successively

‘NS’ here means ‘not specified’. ^15^ Crick *et al.*’s ([1957], p. 420) term. Gamow and Yčas’ ([1955]) scheme proposed non-overlapping triplets, in contrast to the first ‘combination code’ invented and rejected by Crick ([1955]). ^16^ Crick ([1957b]) articulates a ‘quadruplet code’, which is presumably a refinement of the earlier ‘4-group code’ (Crick [1956]). ^17^ As inferred from the correspondence between Szilard and Crick as well as from Kay’s ([2000]) description. Szilard’s manuscript was unavailable.

Within each of these two groups were further differences, mostly relating to the template units:

- Degeneracy: In ‘degenerate’ schemes (Crick [1955]), more than one type of template unit could specify one type of amino acid; in some degenerate codes all amino acids had the same level of degeneracy (labelled ‘uniform’ in Tables 1–2), in others the level varied between amino acids (‘varying’).
- Attachment: Amino acids were taken to either attach directly to template units (‘direct’ in Tables 1–2) or to attract adaptor molecules that carry certain amino acids (‘indirect’); the fact that some schemes posited direct interactions between template units and amino acids will be significant for the discussion of arbitrariness (Section 5).
- Directionality: In ‘directional’ schemes (Crick [1955]), a given nucleic acid sequence specified an amino acid sequence only if ‘read’ in one direction; similarly, at a lower level, individual template units specified amino acids only if ‘read’ in one direction.
- Chemical identity of the template: The template was thought to consist of DNA, RNA, or a combination of nucleic and amino acids.
- Single/Double: Synthesis was hypothesized to occur on a single or double strand (of nucleic acid).
- One/Both: If nucleic acids served as templates in double-stranded form, then a template unit could include bases either from one strand or from both strands.
- Cardinality: The number of bases per template unit, for example, three RNA bases (triplet).

As can be seen from Tables 1 and 2, all schemes involved claims about the nature of hypothetical entities and interactions that were thought to be part of the mechanism of protein synthesis. Gamow’s ([1954a]) scheme, for instance, posited diamond-shaped holes on the DNA double helix as well as stereochemical interactions between them and incoming amino acids. From Gamow’s proposal, Crick generalized ‘a code with the following properties’: four nucleic acid bases (‘letters’), template units that are overlapping triplets, all triplets specify an amino acid, and all amino acids are specified by at least one triplet (Crick [1955], p. 10). Unlike Gamow’s scheme, Crick’s generalized version did not make assumptions about diamond-shaped holes and the like. Even so, it assumed that the template units that determine an amino acid are base triplets, that the base triplets are overlapping, and so on. Finally, consider Crick *et al.*’s ‘comma-less’ scheme: What Crick ([1957], p. 419) called a ‘physical interpretation’ of this scheme posited a single-stranded RNA as a template, which interacted via hydrogen bonding with trinucleotide adaptor molecules (each of which carrying one amino acid). But even without this physical interpretation, Crick’s comma-less scheme made non-trivial assumptions about the physical components of the mechanism. These assumptions included that there are two sets of components—amino acids and base triplets—; that the base triplets are non-overlapping; and that there is some stereochemical interaction between triplets and amino acids such that the sequence of triplets determines the amino acid sequence.

The three examples show that different schemes provided varying degrees of detail. Crick’s generalized Gamow scheme and his comma-less scheme offered comparatively little detail. They postulated base triplets, for instance, but remained silent on how the triplets were spatially organized. Gamow’s diamond scheme, by contrast, assumed that a triplet forms three of the four points of a diamond-shaped hole (the fourth point consisting of one of the paired-up bases). Furthermore, Gamow’s scheme was committed to a DNA double helix template, whereas Crick’s two other proposals were explicitly neutral about whether the template was RNA or DNA. But even Gamow’s diamond scheme was not fully specified—for example, how exactly the amino acids were to interact with the holes so as to generate a specific fit remained an open question.

In addition to hypotheses about the physical components of protein synthesis, all schemes made claims about the mapping between template units and amino acids. ‘Degeneracy’ (Crick [1955], p. 5), for example, referred to a set-theoretic relation between the sets of template units and amino acids: every element of the co-domain (set of amino acids) was mapped to by at least one template unit; the relation was thought to be non-injective and surjective. Crick’s comma-less system assumed a non-functional relation in which only twenty of the sixty-four template units map to the twenty amino acids. An important property of these relations was that they could be shared by otherwise very different schemes. The triangular and dyad scheme, for instance, shared the degenerate type of mapping (see Table 1). Similarly, Crick *et al.* ([1957]) argued that the comma-less scheme was not just one particular scheme but comprised a large class of distinct schemes (they calculated 288) that shared the non-functional relation between twenty triplets and twenty amino acids, but differed with respect to the identity of the twenty triplets.

So far I have used the neutral term ‘scheme’ for these proposals. This term was in fact often employed at the time (Crick [1955], pp. 3, 9), not least by biochemists and biophysicists who were not among Crick’s or Gamow’s immediate co-workers (Dounce [1955]; Schwartz [1955]; Wilkins [1957]). However, ‘scheme’ was just one of several labels used to denote the proposed solutions to the coding problem between 1953 and 1958. Another, less frequently used, term was ‘assignment’ (Dounce [1955]). Most interesting for the purposes of this article was the term ‘code’. This term was used in several different senses, one of which was as a synonym for ‘scheme’ or ‘assignment’.^4^ The first usage of ‘code’ in this sense appears in undated documents from 1953 to 1954 by Crick (for example, ‘Diamond code’ (Crick [1953–4], p. 3)) and in Gamow’s letters in the first half of 1954 (for example, ‘code of triangles’ (Gamow [1954d]). Both authors used the term ‘code’ regularly, though not exclusively. In print, Gamow initially wavered between more neutral terms and the semantically loaded ‘code’. At times he referred to ‘the proposed scheme’ (Gamow [1954b], p. 6) and a ‘unique correspondence’ and ‘translation procedure’ (Gamow [1954a]). In late 1954 Gamow moved from a ‘unique coding procedure’ to ‘the code in question’ (Gamow and Metropolis [1954], p. 779).^5^ So the code metaphor was clearly employed many years before the actual code began to be elucidated in the early 1960s, as has been pointed out before (Judson [1996]; Kay [2000]). But it is worth emphasizing not only that the protagonists of the theoretical approaches to protein synthesis used the term in the 1950s, but also that they employed this term in a specifically scientific context, namely, when thinking about their research and communicating their ideas among themselves. Crick employed ‘code’ for personal record keeping, as his notebooks on Gamow’s scheme testify (Crick [1953–54]). The term also figured in the informal and semi-formal discourse among Gamow, Crick, and their collaborators: it appears in their private letters, which communicated, alongside the gamut of personal news, the latest research activities (for example, Gamow [1954c], [1954d], [1954e]); and it was used when articulating preliminary results and ideas for internal discussion (for example, Crick [1955]; Drake and Alderson [1956]). Its appearance in official publications was but the outwardly visible aspect of its use.^6^

## 3 The Code Concept Played Descriptive and Explanatory Roles

The previous section described how the code metaphor was used by scientists like Crick and Gamow in the 1950s. They began using the term ‘code’ in the context of early research into protein synthesis, especially for the purposes of internal communication and personal record-keeping. In conjunction with prefixes like ‘diamond’, the term picked out a variety of hypothetical schemes that addressed a specific aspect of the mechanism of protein synthesis.^7^ From the way these scientists actually employed the code concept, it is apparent that it referred to several distinct, hypothetical schemes. Furthermore, each scheme postulated hypothetical, physical components, and a mapping relation between template units and amino acids. The components and relations were ordinary physical entities (for example, molecules), processes (for example, chemical interactions), and structural features (set-theoretic relations). There was no indication that the schemes involved any semantic properties (but see below for similarities with human symbol systems). Hence, although the code concept was never formally defined, it was employed in scientific discourse with a definite, albeit variable, content. It was a quasi-technical concept with a clear descriptive-denotational role.

In addition, the hypothetical mechanisms could explain, in outline, how sequences of four nucleic acid bases might specify sequences of twenty amino acids. The coding schemes were, after all, potential solutions of the coding problem. More specifically, coding schemes offered mechanistic explanations because they cited the central causal components and relational features that together were thought to be responsible for generating amino acid chains. And they were possible explanations because the descriptions specified how the outcome might be produced (and they all turned out to be false). Despite omitting many details about the nature of the components and their interactions, the coding schemes were informative. They were sufficiently specific so as to be distinct from other how-possibly explanations, such as those hypothesizing that amino acid sequences are determined by a set of enzymes. Furthermore, scientists like Crick, Gamow, and Brenner explicitly presented coding schemes as solutions to the coding problem and had thus recognized their explanatory value. The codes of the 1950s thus served as how-possibly explanations.

The descriptive and explanatory roles of the code metaphors can be sharpened by distinguishing between mechanism schemas and sketches (Machamer *et al.* [2000]). A mechanism schema is a (propositional or pictorial) representation of a fully known mechanism, describing its various components and how they are organized so as to generate a mechanism’s outcome from its starting conditions. By contrast, mechanism sketches are representations of more or less unknown mechanisms; they are incomplete and sometimes false. It is easily seen that the various codes were descriptions of a mechanism because they described hypothetical components and organizational features of the mechanism of protein synthesis; and they were mechanism sketches because they omitted many details and many of those not omitted turned out to be false. Furthermore, mechanism sketches can vary in the amount of detail they provide (Craver and Darden [2013]). This feature captures an important source of variation among coding schemes. As we saw, some schemes were more specific than others (cf. diamond code versus comma-less code). I will return to this point in Section 4. Finally, the idea of mechanism schemas or sketches is useful because it reminds us that mechanisms do not merely consist of physical components and their interactions, but that they are also organized in a specific way. The mapping between template units and amino acids was the main organizational feature of codes. A mapping like degeneracy, for instance, was independent of the kind of template unit posited by a coding scheme and was thus shared by several schemes. I take these findings to extend Darden and Craver’s ([2002]) previous mechanistic reconstruction of early work on protein synthesis. Darden and Craver focused on molecular biologists’ changing views about the main components of protein synthesis—especially the status of RNA(s) and the chemical bonds involved in ordering amino acids—arguing that their views amounted to mechanism sketches. I agree that their views were sketches. However, Darden and Craver did not discuss the nature of coding schemes (apart from the diamond code) and thus left open the relation of these schemes to mechanisms.

We can now put the conclusion from the foregoing analysis of actual usage as follows: The coding schemes of the 1950s were mechanism sketches, that is, descriptions of hypothetical components and organizational features of one important aspect of the mechanism of protein synthesis. By picking out these schemes in the context of scientific discourse, the code concept played a significant descriptive and explanatory role for the scientists. In the first instance, this is a historical-descriptive claim about the roles ‘code’ played in the 1950s. But there is also a conceptual-normative claim here, namely, that employing the code metaphor was justified and reasonable at least partly because it played these roles. These conclusions are broadly in line with Godfrey-Smith’s ([2000], [2003]) defence of the code metaphor.^8^

## 4 The Abstractness of Codes and the Expendability of the Code Concept

This section considers two worries, one against the historical claim and another against the normative thesis. I will take these in turn.

The idea that the coding schemes were mechanism sketches seems to conflict with a common characterization of coding. Following Crick ([1955]); Crick *et al.* [1957]), the coding problem is often said to be an ‘abstract’ (Judson [1996], p. 248; Sarkar [1996], p. 193) or ‘formal’ (Sarkar [1996], p. 191) problem—that is, a problem distinct from considerations of the mechanistic details of protein synthesis (Judson [1996], p. 248; Sarkar [1996], p. 191). As Sarkar put it:

By this point Gamow had clearly distinguished the abstract coding problem, ‘that of translating a four letter code to a twenty letter code’ [(Gamov *et al.* [1956], p. 24)], from that of finding the mechanism of translation. (Sarkar [1996], p. 193)

It is natural to understand such characterizations of codes and the coding problem as asserting a contrast between coding, on the one hand, and the mechanistic aspects of protein synthesis, on the other hand. To the extent that Crick, Gamow, and others were concerned with coding, they were not concerned with the mechanism. It will then seem unreasonable to think of the coding schemes as mechanism sketches. Yet a closer look at Crick’s and Gamow’s work reveals that there is in fact no tension and that even the most abstract coding schemes were descriptions of mechanisms.

The main source for claims about the abstractness of the coding problem is Francis Crick:

This problem [the ‘coding problem’] is a formal one. In essence it is not concerned with either the chemical steps or the details of the stereochemistry. It is not even essential to specify whether RNA or DNA is the nucleic acid being considered. (Crick *et al.* [1957], p. 416)

Two years earlier Crick maintained that

Gamow’s scheme is essentially abstract. It originally paid lip-service to structural considerations, but the position was soon reached when ‘coding’ was looked upon as a problem in itself, independent as far as possible of how things might fit together. (Crick [1955], p. 6)

For Crick at least, both the coding problem and its solution were abstract. While Crick contrasted coding with structural considerations and stereochemistry, he did not explicitly identify the specific features that made coding abstract. They can be gleaned, however, from Crick’s account of the coding problem and the generalized Gamow code.

According to Crick, the coding problem centres around the mapping of four types of things to twenty types of things (see his quote in Section 2.1). Such mappings are relations between two sets, which can be described by means of set-theoretic notions like surjectivity. Since these features are the subject matter of a formal theory, it is reasonable to regard them as formal or abstract. Another point Crick *et al.* ([1957]) emphasized was the irrelevance of whether or not the template is RNA or DNA. This is a theme that can be traced back to Crick’s generalized version of Gamow’s code (Crick [1955]). Crick’s version included degeneracy, an organizational feature, thus contributing to its abstractness. But the generalized version also makes claims about components, especially about the template units (for example, that they are overlapping triplets; see Section 2.2). As noted above, Crick developed the generalized version by abstracting away from some of the assumptions associated with Gamow’s diamond code, rendering it less detailed. The low-detail character of Crick’s generalized version is another feature contributing to its abstractness.

In conclusion, both the coding problem and its solution(s) involved (i) a mapping between template units and amino acids, and thus a structural property, as well as (ii) components that were described at varying levels of detail. I suggest that these two features constitute (at least partly) the codes’ purported abstractness. That is, coding schemes were abstract to the extent that they included structural features and characterized the components at a low level of detail. Crucially, neither source of abstractness conflicts with mechanistic considerations. Quite the opposite: First, organizational features like the mapping between templates units and amino acids are structural aspects of the mechanism of protein synthesis; they are not something extraneous to the mechanism. Second, low-detail descriptions of triplets and so on are descriptions of components of the mechanism of protein synthesis; such descriptions are not concerned with something other than the mechanism. The apparent conflict between the abstractness of coding schemes and their mechanistic commitments is thus dissolved: the coding schemes of the 1950s were mechanism sketches, and they were abstract insofar as they included structural properties and low-detail descriptions of components.

A second worry concerns the normative claim that the descriptive and explanatory roles of the code metaphor justified its use. ‘Code’ was just one of several terms used in order to refer to the mapping schemes. There was also more neutral vocabulary, like ‘scheme’, ‘correspondence’, and ‘assignment’. This suggests that there is some arbitrariness in choosing the term ‘code’ for these schemes. This point was made forcefully by Lily Kay, who argued that the use of ‘code’ was to a significant extent historically contingent, prompted by the widespread use of crypto-analysis during WWII, the subsequent rise of information technologies, and the personal connections of researchers like Gamow with the US military (Kay [2000], pp. 2, 329). From this point of view, the code metaphor offers no more than an auxiliary label. Its contribution was expendable, not substantial. Some philosophers have advanced arguments to the same effect, albeit not specifically against the code metaphor. These arguments are based on the possibility of replacing semantic with causal language. For instance, Weber ([2005]) doubts the usefulness of the notion of positional information by arguing that its content can be articulated in purely causal terms; choosing informational language to describe and refer to that content does not add anything useful.^9^ Similarly, Šustar ([2007]) re-interprets the central dogma in causal terms and emphasizes that genetic information can be given a purely causal interpretation. From this he concludes not only that the case for a semantic interpretation of genetic information is weakened, but also that the notion of genetic information can be shown to be superfluous. While these objections were not targeted specifically against the genetic code, they can easily be extended to it given the analysis of actual usage presented in this article. For ‘code’ picked out certain causal and structural properties, and there was considerable flexibility in whether or not they were referred to by ‘code’ or by more neutral, non-semantic terms. Considerations like these can seem to undermine the theoretical value of the code metaphor. After all, how significant can the metaphor be if it was expendable?

We should distinguish between several issues here. One question is whether the code concept, as it was or is used in molecular biology, involved semantic commitments in some sense. The historical findings of this article strongly suggest that it does not, at least not in the research context examined here. Sarkar’s ([2003]) assertion that considerations about semantic properties are alien to molecular biology is thus substantiated with respect to coding. I also agree with the spirit of Weber’s and Šustar’s objection: claims about the code involved ordinary causal and structural commitments, and in this sense they did not add anything extra. However, in contrast to Weber and Šustar, my argument for this conclusion does not hinge on the success of any particular causal reconstruction of the causal and structural properties denoted by ‘code’, nor does it rely on the socio-political arguments advanced by Kay ([2000]). I take the conclusion to follow from how the code metaphor was used in practice.

A second issue concerns the extent to which the code played a substantive theoretical role in molecular biology. This is a separate issue because, from the fact that an apparently semantic concept (like code) did not entail semantic commitments, it does not follow that without them it played no useful and substantial role. It might have played roles that did not rely on attributing semantic properties. Furthermore, the historical findings suggest that the code metaphor did play such a role: it referred to a set of causal and structural properties that could solve the coding problem. That is, ‘code’ was descriptive and explanatory. And since the degree to which a scientific concept plays a useful and substantial role depends partly on the degree to which it is descriptive and explanatory, the code metaphor was useful and justified, at least to the extent it played such roles. Lastly, the fact that alternative concepts like ‘scheme’ and ‘assignment’ were used to the same effect is consistent with the code concept also playing such roles.

Finally, there is the worry that if a semantic concept does not add anything extra, in particular nothing semantic, then using a semantic concept is obsolete and even potentially misleading. It suggests semantic commitments where there are none. In other words, the worry is that semantic language is spurious and less appropriate when referring to ordinary causal or structural properties. Accordingly, one might concede that ‘code’ played descriptive and explanatory roles (by referring to mapping schemes), while still maintaining that it was inappropriate to construe the process as one of coding. This is a reasonable concern. And yet, as I will now argue, the actual usage suggests that the code concept was used appropriately and that it was in fact better suited for these roles than more neutral notions.

Not any mapping between two sets qualifies as a conventional code or cipher. Conventional codes are rather more specific kinds of mappings: among other peculiarities, they typically relate the elements of two symbol sets such that we can produce one string of symbols from the other (for example, an English sentence from a message in Morse signs). Indeed, the ability to generate one type of symbol sequence from another is one the central purposes of conventional codes and ciphers. The coding schemes of the 1950s shared this paradigmatic feature. The mappings that were hypothesized as part of these schemes posited a set of relations between entities such that one kind of sequence can be generated by means of another. Achieving this feat was thought to be the main contribution that these relations made to protein synthesis (Section 2.2). Coding schemes did not posit set-theoretic relations between elements that were not causally connected in this way. By transferring the term ‘code’ from the source domain of human symbol systems to molecular biology, it was possible to exploit this particular analogy. The code metaphor conveyed the idea that the molecular mapping was not just any kind of mapping, but rather a mapping by which one kind of sequence can be generated by means of another—that is, a kind of mapping that could solve the coding problem. For this reason, the term ‘code’ was an apt choice. Furthermore, the code concept was more appropriate than neutral concepts like ‘system’, ‘scheme’, and ‘assignment’. The latter notions entail, at best, that there is a mapping between elements, but not that the mapping enables the generation of one kind of sequence from another. Since they could not convey this critical feature, they were less well suited for picking out the molecular relations in question. Replacing ‘code’ with a non-semantic notion would thus have incurred a cost.^10^

## 5 The Role of Arbitrariness

In this last section I consider the significance of arbitrariness for understanding the role of coding schemes. According to an influential line of thought, the arbitrariness of human symbol systems is analogous to the absence of a ‘chemical necessity’ between the codon-amino acid assignments of the genetic code (Godfrey-Smith [2000]; Maynard Smith [2000]). Human symbol systems like the Morse code are arbitrary insofar as the form of a Morse symbol is not tied to its meaning (Maynard Smith [2000], p. 185). The physical properties of the symbol ‘• • •’, for example, do not compel the dots to stand for ‘S’; nor do they prevent the dots from being assigned to a different letter. Analogously, the argument goes, the physico-chemical properties of any given codon do not restrict it to specify only the amino acids it presently specifies: if the tRNAs complementary to a given codon changed their stereochemical properties so that they became complementary to an aminoacyl-tRNA synthetase that carries a different amino acid,^11^ then the same codon would end up specifying a different amino acid. Several authors have argued that this analogy is an important motivation for the code concept (Godfrey-Smith [2000]; Maynard Smith [2000]; Kjosavik [2007]; Barbieri [2008]). The intended content and scope of this claim remain somewhat unclear. It can be interpreted historically, for example, as a claim about why scientists like Crick and Gamow began using the term ‘code’. It could also be taken normatively, as providing a rationale or justification for its use. However, neither claim seems plausible in the light of the present findings about actual usage.

Consider first the historical claim that the code metaphor was introduced and/or employed in the 1950s at least partly in order to highlight the analogy with the arbitrariness of conventional symbols. This claim is implausible. First, I found no evidence that arbitrariness of human symbol systems was discussed or investigated between 1953 and 1958, either in connection with direct or with indirect coding schemes. Second, the early schemes were incompatible with arbitrariness because they posited direct assembly of amino acid chains on templates. In a direct assembly scheme, a given template unit can specify a different amino acid only if it is assumed that the unit changes its stereochemical properties so as to become directly complementary to a different amino acid; the unit’s functional groups would need to be modified, added, or eliminated. Since any such change would alter the units’ molecular structure, it would cease to be the same unit as defined by that structure. It was thus impossible for the units of early, direct assembly schemes to specify an amino acid other than the one it was taken to specify. In other words, direct assembly schemes excluded the possibility of arbitrariness. It is thus hard to see how arbitrariness could have motivated the construal of these schemes as codes.

Let us turn to the normative claim, which comes in different strengths. A comparatively weak claim is that arbitrariness contributes to the justification of the code metaphor (for example, Maynard Smith [2000]). A stronger claim is that arbitrariness is crucial, perhaps even necessary, for its justification. The stronger view seems to underpin a thought experiment by Godfrey-Smith ([2000], [2003]). Godfrey-Smith invites us to imagine a world in which protein synthesis proceeds without coding and then asks whether, if coding were absent, other features of an organism’s biology would need to be different as well. Here I focus not on the outcome of the thought experiment, but rather on the idea of protein synthesis without coding. According to Godfrey-Smith, there would be no coding if the template was an amino acid chain and incoming amino acids paired up like-with-like. Such a mechanism would involve neither arbitrariness nor two distinct classes of molecules (since both template and product would be proteins), and it is presumably for these reasons that Godfrey-Smith deemed it inappropriate to construe the mechanism as a coding process. So, on this view, arbitrariness seems to be a crucial component in justifying coding talk. The idea is perhaps that arbitrariness is so central to human symbol systems that its absence in the biological system would render the similarity superficial and, hence, the code metaphor poorly motivated. As a consequence, the scientists who construed direct assembly schemes as coding processes would have been misguided.

The actual usage of the code metaphor suggests, however, that there is no compelling reason to hang so much weight on arbitrariness. Admittedly, the lack of arbitrariness in some coding schemes does constitute a disanalogy with human symbol systems. But disanalogies abound and do not by themselves undermine the power of an analogy. The coding metaphor could serve to highlight other similarities that are as salient as arbitrariness. In fact, there is an important similarity that commentators have so far overlooked: human symbol systems and the hypothetical template amino acid assignments are both instances of mapping relations that enable one sequence of entities to be generated from another (as argued in the previous section). Since the scientists’ primary goal was to understand how nucleic acid sequences could determine amino acid sequences (the ‘coding problem’), it is not difficult to see that the code metaphor emphasized this analogy. Furthermore, the fact that both direct and indirect assembly schemes were construed as coding processes indicates that the similarity in generating sequences outweighed the disanalogy arising from arbitrariness.

The value and legitimacy of scientific concepts depends at least partially on their theoretical and heuristic usefulness. The code metaphor did well on this benchmark, and it did so even without arbitrariness. Schemes without arbitrariness were just as explanatory as schemes with arbitrariness (cf. diamond versus comma-less code). And schemes lacking arbitrariness did not in any way have less of a descriptive-denotational role. So the code metaphor was justified to a significant extent by its descriptive and explanatory roles, and these roles were independent of arbitrariness.

## 6 Conclusions

This article reconsidered the legitimacy of the code concept in molecular genetics. I analysed how the code metaphor was actually employed by the scientists who first promoted its use in the 1950s. The analysis showed (i) that the term ‘code’ had multiple referents, namely, schemes that accounted for how nucleic acids might determine amino acid sequences; (ii) the schemes were mechanism sketches, consisting of more or less detailed, and often false, descriptions of ordinary molecular components, processes, and structural properties of the mechanism of protein synthesis; (iii) none of the schemes included recognizably semantic properties, and many early codes excluded the possibility of arbitrariness; and (iv) it played at least two valuable theoretical roles, namely, describing and designating mechanism sketches by exploiting a specific analogy with human symbol systems, as well as providing how-possibly explanations for the ordering of amino acids by nucleic acids.

These conclusions are compatible with the view that the code is essentially ‘abstract’. I argued that coding schemes can be construed as abstract to the extent that they provided low-detail descriptions of components and emphasized structural features of the mechanism of protein synthesis. Furthermore, the fact that more neutral terms like ‘scheme’ were also used to pick out the same mechanism sketches does not undermine the appropriateness of using the semantically loaded concept of code. Its justification stems from its theoretical roles and from highlighting a distinct similarity with human symbol systems.

At some point after 1958, leading researchers like Crick emphasized the purportedly accidental nature of the genetic code and thereby invited analogies with the arbitrariness of human symbol systems. But arbitrariness played no discernible role in the development of early coding schemes and was in fact incompatible with many of them. Historically, arbitrariness was an idea added after the code had become established among molecular geneticists. And, from a philosophical point of view, arbitrariness is unnecessary for justifying the code concept in molecular biology.

## Footnotes

^1^ The documents of well-known figures like Crick and Watson are better represented in online archives, which is likely to have introduced a bias. For practical reasons, this limitation was deemed acceptable. Furthermore, these researchers were the main users of the code concept and are thus the natural focus of any attempt to identify its actual usage.

^2^ For molecular biologists in the 1950s, ‘determination’ expressed a kind of exclusive causal dependence of the amino acid order on the nucleic acid order. A further explication of their notion of determination is beyond the scope of this article, as is an assessment of whether it amounts to a sound causal notion. For attempts in this direction, see (Waters [2007]; Woodward [2010]; Stegmann [2014]).

^3^ Watson and Crick drew up the list of twenty naturally occurring amino acids when they received Gamow’s manuscript in the summer of 1953 (Judson [1996], p. 258).

^4^ The word ‘code’ was also employed in other ways, for instance, it was used interchangeably with a base sequence (Watson and Crick [1953b], p. 965) or an ‘alphabet’—for example, the set of twenty different amino acids (Gamow *et al. *[1956], p. 24). This article is only concerned with ‘code’ in the mapping sense.

^5^ Sarkar argues that Gamow’s use of ‘code’ in 1954 can be traced back directly to Schrödinger’s ([1944]) ‘codescript’ (personal communication, cf. Sarkar [2013]).

^6^ The schemes listed in Tables 1 and 2 were those explicitly labelled ‘code’ between 1953–58. Other schemes published during this time (Dounce [1955]; Schwartz [1955]) were not referred to as ‘codes’, by their inventors or by commentators such as Stent ([1955]) and Crick ([1957c]).

^7^ The arguments in this article do not depend on any particular account of mechanism and thus remain neutral on this point (for discussion see, for example, Machamer *et al. *[2000]; Glennan [2002]; Torres [2009]).

^8^ Godfrey-Smith ([2003]) argued that the code concept is justified because (i) it picks out features that explain the linear ordering of amino acids during protein synthesis and because (ii) these features exhibit three specific similarities with human symbol systems. In contrast to Godfrey-Smith’s account, my conclusions are based on an analysis of actual usage, which reveals that arbitrariness was not one of the intended positive analogies with human symbol systems (see Section 5).

^9^ Weber ([2006]) offers a causal construal of the central dogma. In his ([2005]), however, he was careful to point out that the possibility of a causal reconstruction does not imply that ‘code’ or ‘information’ do no significant work.

^10^ This should be understood as a claim about the factual benefit of the code metaphor, rather than a claim about the intentions of molecular biologists. I do not maintain that they deliberately exploited the metaphor for this purpose. This is because there are few sources that reveal their motivation for introducing and using the code metaphor. Perhaps the most explicit remark about the motivation at the time was Watson’s observation that protein synthesis bears ‘obvious analogies to conventional coding problems in which we go from one language to another’ (Crick *et al.* [1954–55]). He did not specify the ‘obvious analogies’. But he described ‘conventional coding problems’ as going from one language to another, thus suggesting a process of translating strings of symbols from one language to another. And in such a process, coding relates the elements of the languages such that one can produce one symbol string from the other. So the argument above is at least compatible with Watson’s remark.

^11^ These are the enzymes that catalyse the attachment of amino acids to their specific tRNAs.
